# Association between sarcopenia and survival in patients with gynecologic cancer: A systematic review and meta-analysis

**DOI:** 10.3389/fonc.2022.1037796

**Published:** 2023-02-20

**Authors:** Wen-Li Lin, Thi-Hoang-Yen Nguyen, Cheng-Yao Lin, Li-Min Wu, Wen-Tsung Huang, How-Ran Guo

**Affiliations:** ^1^ Center for Quality Management, Chi Mei Medical Center, Liouying, Tainan, Taiwan; ^2^ School of Nursing, Kaohsiung Medical University, Kaohsiung, Taiwan; ^3^ Department of Environmental and Occupational Health, National Cheng Kung University, Tainan, Taiwan; ^4^ Division of Hematology and Oncology, Department of Internal Medicine, Chi Mei Medical Center, Liouying, Tainan, Taiwan; ^5^ Department of Medical Research, Kaohsiung Medical University Hospital, Kaohsiung, Taiwan; ^6^ Department of Occupational and Environmental Medicine, National Cheng Kung University Hospital, Tainan, Taiwan

**Keywords:** sarcopenia, gynecologic cancer, survival, mortality, systematic review, meta-analysis

## Abstract

**Background:**

Despite prior attempts to evaluate the effects of sarcopenia on survival among patients with gynecologic cancer, the results of these studies have not been consistent. The present study evaluated the association between sarcopenia and survival among patients with gynecologic cancer by aggregating multiple studies.

**Methods:**

We performed a literature search using computerized databases and identified additional studies included in the bibliographies of retrieved articles. The quality of each study was evaluated using the Newcastle–Ottawa Scale, and meta-analyses were performed to evaluate overall survival (OS) and progression-free survival (PFS). We constructed a forest plot for each outcome and assessed publication bias using Begg’s test. Heterogeneity was assessed using I^2^ statistics.

**Results:**

From the 5,933 initially identified articles, 16 studies describing 2,031 participants with a mean age of 60.34 years were included in the meta-analysis. We found that compared with patients with gynecologic cancer but without sarcopenia, patients with sarcopenia had worse OS, with a pooled hazard ratio (HR) of 2.61 (95% confidence interval [CI]:1.52–4.46), and worse PFS (HR: 1.37, 95% CI: 1.09–1.73). The quality of studies was generally good, and no publication bias was detected among studies for either OS or PFS. Although 4 of 12 studies were of fair quality, we conducted a sensitivity analysis excluding studies or fair quality and obtained similar results.

**Conclusions:**

These meta-analysis results suggest that sarcopenia is associated with worse OS and PFS among patients with gynecologic cancer. The use of different case definitions appeared to be a major source of heterogeneity among the studies. Further studies remain necessary to confirm our findings, especially those examining OS and PFS, because publication bias was identified.

## Introduction

Gynecologic cancers refer to the five primary forms of cancer that affect a woman’s reproductive organs: cervical, ovarian, uterine, vaginal, and vulvar cancers. Cervical cancer is the fourth most common cancer diagnosed among women worldwide and was the leading cause of cancer-related deaths in women in Eastern, Western, Central, and Southern Africa in 2018. Globally, the average age at cervical cancer diagnosis is 53 years, and the global average age at death is 59 years (1). Despite tremendous progress, 14,100 women in the United States are estimated to be newly diagnosed with cervical cancer in 2022, with 4,280 women dying as a result of their disease (2). Ovarian cancer is the seventh most common cancer in women and the eighth-most common cause of cancer-related death, with 5-year survival rates below 45% (3). High surgical complexity carries an inherent risk of postoperative complications, including anastomosis leakage. Preoperative nutritional status and surgical characteristics, such as a body mass index of < 18 kg/m^2^, preoperative albumin level of < 30 mg/dL, section of the inferior mesenteric artery at its origin, and medium to low colorectal anastomosis, have been identified as independent risk factors of anastomosis leakage (4). Chemotherapy, intraperitoneal chemotherapy, immunotherapy, and targeted therapies are potential therapeutic options under study to improve the outcomes of gynecologic cancer treatment (5–7). In the field of personalized medicine, researchers attempt to identify novel therapeutic targets, and the patients’ physical ability to receive therapy is an important consideration. One contributor to a patient’s ability to receive therapy is their muscular mass. Low muscle mass, also known as sarcopenia, has been linked to worse prognosis in a variety of cancers, including pancreatic (8, 9), hepatic, biliary tract, gastrointestinal (10), and lung cancers (11). It also has an important role in ovarian cancer patients’ outcomes (12), especially when it is noted at baseline (13). Sarcopenia occurs during the natural aging process, resulting in the loss of strength with age (14). Although the gold standards for non-invasive muscle quantity/mass evaluation are magnetic resonance imaging and computed tomography (CT), the cutoff criteria for determining low muscle mass have not yet been firmly established (15). A number of studies have identified sarcopenia as a predictive factor for survival in patients with gynecological cancer (16, 17). However, other studies have found no link between muscle loss and survival in women with gynecological cancer (18, 19). By combining multiple study outcomes, the current study assessed the relationship between sarcopenia and survival among patients with gynecologic cancer.

## Materials and methods

### Literature search

We searched PubMed, Embase, Scopus, and the Cochrane Library for potentially relevant publications, without regard to publication date or language. The subject headings and search structures of each database were used to tailor search tactics. Reference lists from review articles were also searched for potentially relevant publications. To achieve the comprehensive retrieval of relevant studies, we employed the following keyword search strategy: (uteri* OR uterus* OR womb OR endometri* OR ovarian OR ovary OR cervical OR cervices OR vulva* OR vaginal OR vagina) AND (cancer OR carcinoma OR tumor OR neoplasm) AND (muscular atrophy OR sarcopenia OR sarcopenia OR skeletal muscle depletion OR muscle index OR muscle mass). The search was completed on April 1, 2022.

### Inclusion and exclusion criteria

All identified studies were evaluated for the following inclusion criteria: the study examined sarcopenia and outcomes in any gynecologic cancer; participants were women with uterine/endometrial, ovarian, cervical, vulvar, or vaginal cancer; an approved objective measure was used to define sarcopenia; and the study design was a randomized controlled trial or cohort study (both prospective and retrospective). The following exclusion criteria were applied: abstracts conferences, case studies, animal studies, review studies, management recommendations, and pharmaceutical therapy trials; studies that did not report on pre-determined outcome measures of interest; and studies that did not characterize.

### Data extraction

After removing duplicates, two researchers independently assessed the titles and abstracts of all articles identified during the literature search. The full text of potentially relevant articles was obtained for further review. Any disagreements among reviewers regarding a study’s eligibility were resolved through a conversation with a third senior author.

### Quality appraisal

We adapted the Newcastle–Ottawa Scale (18) for evaluating study quality, as the application of this scale to evidence-based reviews and meta-analyses has been shown to produce highly objective results. The Newcastle–Ottawa Scale evaluates several domains, including selection, comparability, and outcome measurement. All studies were independently scored in each domain by the two co-authors. Consensus was reached on the classification of each study by comparing the results of the individual researcher.

### Meta-analysis

Overall mean effect sizes were estimated using either random-effects or fixed-effects models, depending on the heterogeneity identified among the included studies, which was assessed using the I^2^ statistic (fixed-effects models were used when I^2^ < 50%) (19, 20). The multivariate survival analysis in each study was selected for analysis. If a study did not include multivariate survival analysis, univariate analyses were used.

Sensitivity analysis was performed by excluding studies with outlier effect estimates from the analysis. Outliers were identified as those with a 95% confidence interval (CI) that differed from the 95% CI of the pooled effect. Trials with the potential to introduce heterogeneity across studies were also excluded from sensitivity analyses. After excluding studies with potential heterogeneity, the overall effect was recalculated. To evaluate publication bias, we used funnel plots and Begg’s test.

All statistical analyses were performed using RStudio Version 1.3.1093. The “metafor” package was applied to conduct meta-analyses. Significance was defined as a two-tailed p-value < 0.05.

## Results

**Figure 1:**
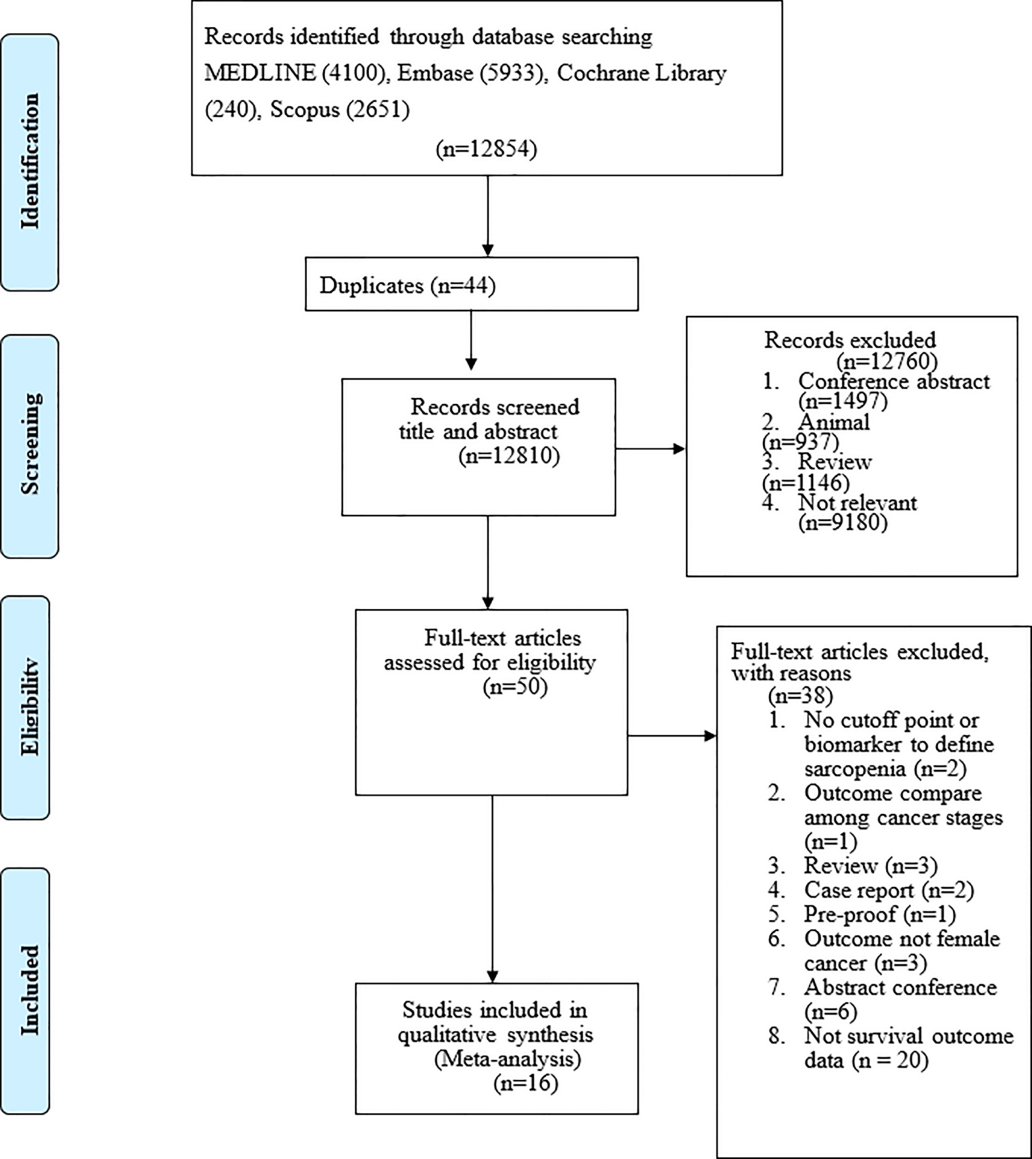
Flow diagram of the literature search.

After eliminating duplicate studies, 12,854 publications were identified during the electronic database search. After a title and abstract review, 12854 publications were excluded, leaving 44 publications (**Figure 1**). Of these 50 publications, we excluded studies that used no cutoff point or biomarker to define sarcopenia (n = 2); studies with outcomes compared across cancer stages (n = 1); review articles (n = 3); case reports (n = 2); study protocols (n = 1); studies that did not examine patients with gynecologic cancer (n = 6); abstracts (n = 6); and studies that did not report survival outcomes (n = 14). Finally, 16 articles were included in the meta-analysis (**Table 1**), including 2,031 participants with a mean age of 60.34 years. No additional studies were identified from the bibliographies of retrieved articles. All 16 studies had a score of 6 or higher, indicating that they were of moderate to high quality (**Table 1**).

**Table 1:** Summary of the included studies.

Reference	Number/Age (mean)	Cancer type/FIGO stage	Sarcopenia Criteria Cutoff (prevalence)	Progression-Free Survival	Overall Survival (HR, 95%CI)	Newcastle-Ottawa scale
Aust et al, 2015 (19)	N=140/60	Ovarian/I-IV	SMI 41cm^2^/m^2^ (28.9%) (CT scan at L3 level)	1.31 (0.76–2.26)	1.23 (0.61–2.48)	8
Bronger et al, 2017 (13)	N=128/63	Ovarian/III-IV	SMI 38.5 cm^2^/cm^2^ (11%) (CT scan at L3 level)	2.52 (1.10-5.81)	2.89 (1.11–7.54)	7
Chae et al, 2021 (21)	N=82/52	Ovarian/I-II	SMI 38.7 cm^2^/cm^2^ (20.7%) (CT scan at L3 level)	–	58.4 (3.02–1,127.9)	8
de Paula et al, 2019 (22)	N=232/64.3	Endometrial/I-IV	SMI 38.9 cm^2^/cm^2^ (25.8%) (CT scan at L3 level)	–	2.23 (1.19–4.20)	7
Ganju et al, 2020 (23)	N=64/61	Endometrial/III-IV	SMI 41cm2/m2 (44%)	–	3.02 (1.04–8.74)	6
Kim et al, 2020 (20)	N = 179/57.5	Ovarian/III-IV	SMI 39.0 cm^2^/cm^2^ (42.5%) (CT scan at L3 level)	1.292 (0.906–1.843)	0.870 (0.488–1.550)	7
Kiyotoki et al, 2018 (24)	N = 60/56.1	Cervical Cancer/I-IV	SMI and IM determined from the mean value 90.29 cm^2^ and 10.07 cm^2^	1.62 (0.53-4.97)	2.89 (0.74-11.24)	6
Lee et al, 2018 (25)	N = 245/63	Cervical Cancer/I-IV	SMI 41 cm^2^/cm^2^ (51.8%) (CT scan at L3 level)	–	6.02 (3.04–11.93)	8
Brooks et al, 2019 (26)	N = 148/54.3	Endometrial/III	SMI 39.3 cm^2^/cm^2^ (33.6%) (CT scan at L3 level)	0.67 (0.27–1.70)	0.67 (0.29–1.52)	7
Matsubara et al, 2019 (17)	N=92/55.3	Ovarian/I-IV	SMA <92.92 cm2 (50%) (CT scan at L3 level)	1.272 (0.725-2.230) (univariate)	2.186 (1.057-4.518) (univariate)	8
Rutten et al, 2017 (27)	150/67	Ovarian/II-IV	SMA > 2%/100 days (NR)		1.698 (1.038–2.778)	6
Rutten et al, 2017 (28)	N =216/63.1	Ovarian/II-IV	SMI 38.73 cm^2^/cm^2^ (32.4%) (CT scan at L3 level)	–	1.362 (0.968-1.916)	6
Rutten et al, 2016 (29)	N=123/66.5	Ovarian/II-IV	SMI 41.5cm2/m2 (67.5%) (CT scan at L3 level)		1.773 (1.018–3.088)	6
Yoshikawa et al, 2020 (30)	N = 40/56.9	Cervical Cancer/IV	PMI: 3.72 cm^2^/m^2^ (CT scan at L3 level)	–	4.55 (1.36–18.21)	6
Yoshikawa et al, 2021 (31)	N = 72/62	Ovarian/I-IV	PMI: 5.4 cm^2^/m^2^ (50%) (CT scan at L5 level)	–	3.87 (1.37−12.1)	7
Yoshino et al, 2020 (32)	N = 60/63.5	Ovarian/I-IV	SMI 39 cm^2^/cm^2^ (60%) (CT scan at L3 level)	–	3.17 (1.18-9.06)	6

FIGO, International Federation for Gynecologic Oncology.

SMI, skeletal muscle index.

IM, iliopsoas muscle.

PMI, Psoas muscle index.

**Figure 2:**
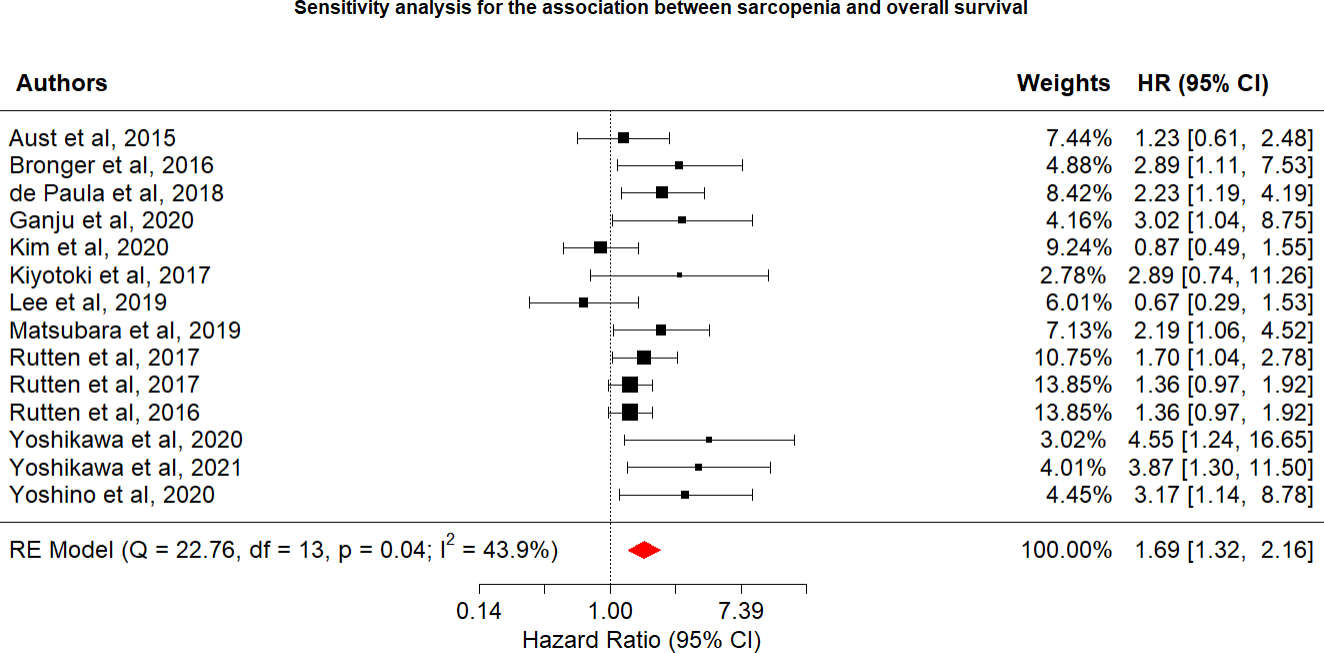
Sensitivity analysis for the association between sarcopenia and overall survival.

Among the 16 included studies reporting the relationship between sarcopenia and OS in women with gynecologic cancer, the overall pooled hazard ratio [HR] was 2.61 (95% CI: 1.52−4.46, I^2^ = 91.0%), with heterogeneity identified among the included studies. In the OS sensitivity analysis, two studies were excluded (20), which reduced heterogeneity below the cutoff value (I^2^ = 43.9%). In the sensitivity analysis, the association between sarcopenia and worse OS was still observed (HR: 1.69; 95% CI: 1.32–2.16; **Table 2**; **Figure 2**). OS and sarcopenia appear to have an inverse relationship, with the presence of sarcopenia associated with a negative impact on OS.

**Table 2:** Hazard ratios and indicators of heterogeneity obtained from the main and sensitivity analyses.

Item	Overall Survival	Progression-Free Survival
Main analyses		
Number of studies	16	6
HR (95% Confidence Interval)	2.61 (1.52−4.46)	1.37 (1.09−1.73)
I^2^ (%)	91.0	0.0
Sensitivity analyses—exclude 2 studies		
Number of studies	14	
HR (95% Confidence Interval)	1.69 (1.32−2.16)	
I^2^	43.9	

**Figure 3:**
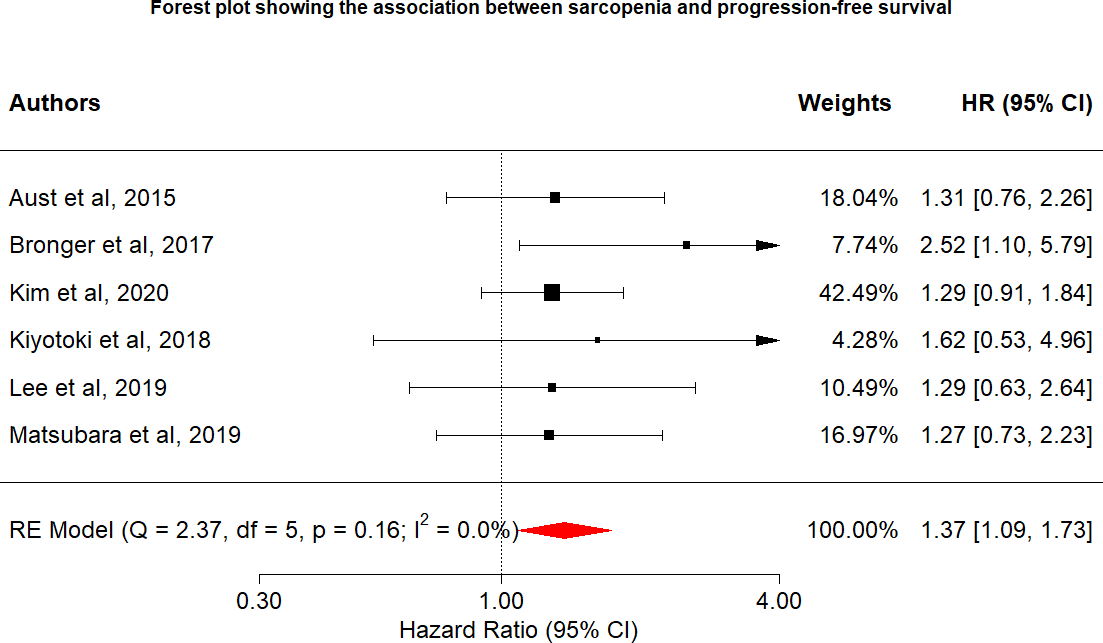
Forest plot showing the association between sarcopenia and progression-free survival.

Overall, six studies included data on the relationship between sarcopenia and disease progression. Overall PFS and PFS according to cancer type are shown in **Figure 3**. Overall, the risk of disease progression increased by 37% among those with sarcopenia (HR: 1.37, 95% CI: 1.09–1.73, n = 6, I^2^ = 0.0%), with no heterogeneity identified among the included studies. The relationship between PFS and sarcopenia is inverse, indicating that the presence of sarcopenia has a negative impact on PFS.

No evidence of publication bias was identified for studies assessing PFS. The Begg’s test indicated publication bias present among studies assessing OS (p = 0.04).

## Discussion

### Associations between sarcopenia and survival outcomes in gynecologic cancer

Recently published articles have updated the definition and diagnostic criteria for sarcopenia, in addition to providing clinical data; however, no established consensus definition of sarcopenia currently exists. **Table 1** summarizes the included studies considered in our present meta-analysis examining the relationship between sarcopenia and gynecologic cancer. We identified 16 studies, which each applied a separate set of criteria for identifying sarcopenia, including 12 studies that defined sarcopenia according to skeletal muscle index (SMI), 2 studies according to skeletal muscle area (SMA), and 2 studies according to psoas muscle (PM) index (PMI). This meta-analysis identified that patients with gynecologic cancer and sarcopenia were associated with significantly worse OS than patients with gynecologic cancer without sarcopenia. Another meta-analysis examined the association between sarcopenia and OS in patients with gynecologic cancer and found similar results (25, 33). However, a separate meta-analysis that limited inclusion to those studies that utilized SMI measurements based on CT scans performed at the L3 level reported a negative association between sarcopenia and OS (33).

Six studies included in our analysis examined PFS, and we found a significant association between sarcopenia and PFS with no evidence of heterogeneity. This finding is inconsistent with the results of other oncological investigations examining the impacts of sarcopenia on PFS (HR = 1.54, 95% CI = 0.90–2.64) (34). A recent meta-analysis pooled four studies and found a positive association with significant heterogeneity across studies (HR =1.69, I^2^ = 54.6%) (35). The significant impact of sarcopenia in patients with specific gynecologic cancers is difficult to investigate due to the small number of studies. However, sarcopenia has the potential to play a role in the progression of ovarian, cervical, and endometrial malignancies, and more research exploring the mechanisms underlying these effects remains necessary. Muscle mass should be viewed not only as a structure indispensable to mobility and vitality but also as a reserve of amino acids, which are essential to severely ill patients.

The depletion of muscle mass has been shown to be associated with poor outcomes, in relation to survival as well as to quality of life and tolerance to oncologic treatments. Skeletal muscle depletion during chemotherapy was found to be associated with poor prognosis, regardless of changes in BMI (4). Muscle mass is not only a structure indispensable to mobility and vitality, but also a reserve of amino acids that are essential to severely ill patients (36); this may be one of the mechanisms of sarcopenia’s link to poor survival. The occurrence of sarcopenia during disease and the mechanisms underlying the possible detriment to prognosis should be better investigated (12).

### Defining sarcopenia

The number of articles examining the effects of sarcopenia is increasing; therefore, a clear and consistent definition of sarcopenia in clinical practice is essential to allow for comparisons across studies and the accurate diagnosis. However, no comprehensive review examining the terminology and methodologies applied to defining or diagnosing sarcopenia has been conducted to date. All of the studies included in our analysis used CT to define sarcopenia, using images obtained at the L3 level, except for one study that used images obtained at the L4 level. Commonly used CT-based sarcopenia indexes include the SMI, PM area (37), PMI, SMA, Hounsfield unit range, and intramuscular adipose tissue content (IMAC). The most common method used to identify sarcopenia among the studies included in our analyses was the SMI (15 studies), followed by PMI (2 studies). Only one study (16) used any other index, and the estimated HR associated with disease-free survival was considered an outlier, indicating the importance of the index used. Even across studies using the same index, the cutoff values used to define sarcopenia differ, influencing the ability to identify associations between sarcopenia and survival.

Sex-specific cutoffs for defining sarcopenia have previously been suggested based on the optimization of stratification methods; however, no official consensus guidelines have been adopted for the cutoff values used to define sarcopenia. Furthermore, most cutoff values were established by studies examining patients from Western societies, and different races and ethnicities are known to have different baseline muscle and fat masses. Among the studies included in our analyses, the cutoff values used to define sarcopenia based on the SMI ranged from 32.5 to 46.6 cm^2^/m^2^ for women and 35.5 to 54.33 cm^2^/m^2^ for men. The most frequently used cutoff value for women was 41 cm^2^/m^2^ (5 studies), followed by 38.5 cm^2^/m^2^ (3 studies). All three studies using the cutoff value of 38.5 cm^2^/m^2^ were published in 2021, indicating a tendency toward smaller cutoff values in recent years. The most frequently used cutoff value for men was 43 cm^2^/m^2^ (5 studies), followed by 53 cm^2^/m^2^ (3 studies), but no decreasing trend in the cutoff values used for men was observed, unlike that in women. Differences in the prevalence of sarcopenia across studies may be due to the use of different indexes and cutoff values to assess the presence of sarcopenia.

## Limitations

**Figure 4:**
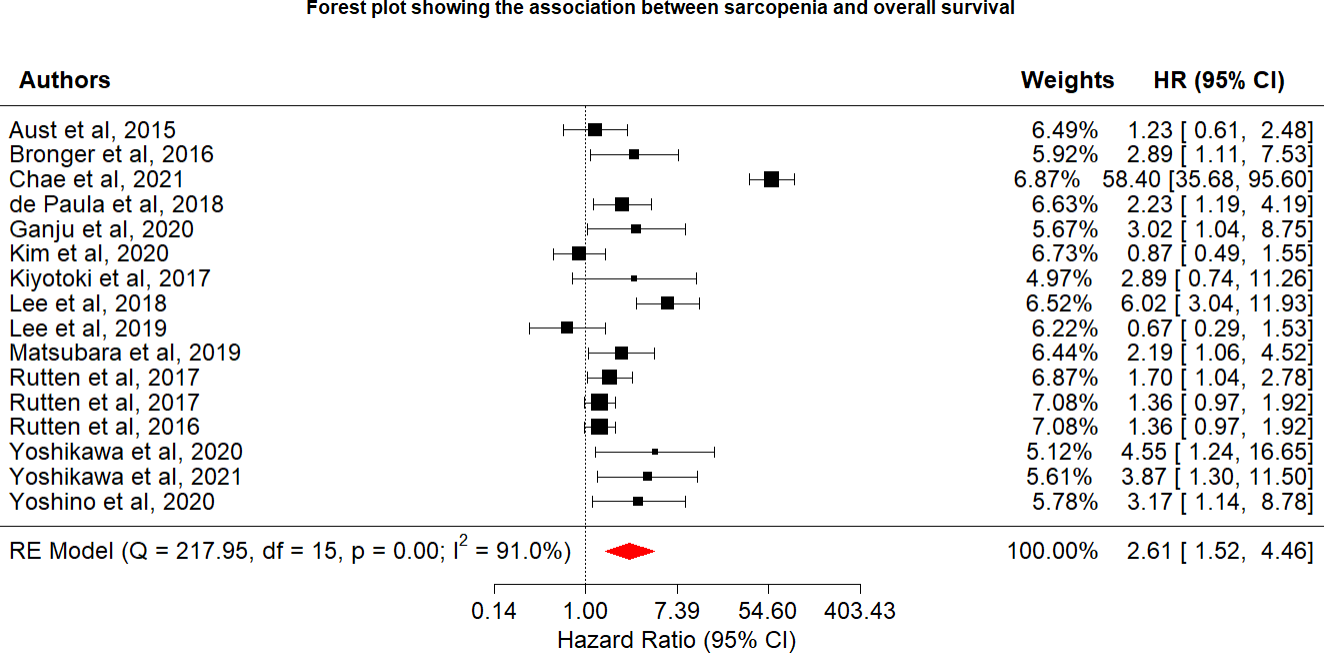
Forest plot showing the association between sarcopenia and overall survival.

Various limitations to this study should be considered. First, we discovered a strong link between sarcopenia and OS, which was consistent with the findings of a recent study (25) that identified sarcopenia as an independent predictor of OS, with 78% increase (HR = 1.78) in OS risk among individuals with sarcopenia than those without sarcopenia, although this study was associated with significant heterogeneity (I^2^ = 78.3%). In the sensitivity analysis for the present study, we removed two studies. The study by Chae et al. (21) was removed because the 95% CI for the OS effect size was an outlier relative to the 95% CI for the pooled effect size (**Figure 4**) (20). In another study, skeletal muscle loss was investigated as an imaging outcome biomarker following final radiation therapy for locally advanced cervical cancer. However, not all patients in this study received the same treatment, and individuals who presented with constant or increasing SMI received more chemotherapy and long-term radiation therapy than those in the reduced SMI group (21). Therefore, the studies’ conclusions regarding the contributions of muscle loss to survival were unclear. After eliminating these two studies, the pooled effect size remained significant (HR = 1.69), with low heterogeneity (I^2^ = 43.9%), indicating that these two studies may have been potential causes of heterogeneity across studies.

The diverse methods used to assess sarcopenia and the retrospective data collection approaches used in the included studies also represent significant limitations of this analysis, as retrospective data collection likely contributed to potential risk of bias, and publication bias was also detected. The accepted definitions and exact measurements used to evaluate sarcopenia differed significantly between investigations, limiting the interpretation of the findings and the ability to systematically compare the included studies.

## Implications for future research

A consensus must be reached regarding the standardized cutoff values used to define sarcopenia in female patients with gynecologic cancers. Future studies should include information regarding muscular strength and nutritional assessments because physical exercise therapies have the potential to prevent sarcopenia and improve physical function among cancer patients.

The majority of existing research examining the link between sarcopenia and gynecologic cancer focuses primarily on OS, with only six of the included studies reporting PFS as an endpoint and only one study reporting recurrence-free survival. No information regarding disease-free survival was reported in any of the included studies. Gynecologic cancer is heterogeneous and diverse, with cervical, ovarian, and endometrial cancers having distinct biologies and risk factors as well as treatments. Therefore, further analyses stratified by treatment type should be performed in future studies on this topic. In the case of the effects on different types of cancer, based on the fact that only 1 out of the 6 estimates of PFS and 2 out of the 12 estimates of OS indicated a better prognosis associated with sarcopenia, we believe that our conclusions are unlikely to change.

The “enhanced recovery after surgery” (ERAS) intervention has been continually developed for many surgical disciplines. The preoperative phase (3–7 days) consists of a prehabilitation concept involving physiotherapeutic exercises and nutritional therapy using a high-calorie protein-rich diet under the supervision of a nutritionist, and improves the surgical outcome of gynecologic cancer (38). Our meta-analysis indicates paying further attention to this concept.

## Data availability statement

The original contributions presented in the study are included in the article/supplementary material. Further inquiries can be directed to the corresponding author.

## Ethics statement

The manuscript is a review article and does not contain clinical studies or patient data.

## Author contributions

Conception and design of study, H-RG. Acquisition of data L-MW and T-H-YN. Analysis and/or interpretation of data W-LL, W-TH and C-YL. Drafting the manuscript, W-LL. Revising the manuscript critically for important intellectual content, H-RG. Data validation, H-RG and L-MW. Supervision H-RG. All authors contributed to the article and approved the submitted version.
